# Among-year variation in the repeatability, within- and between-individual, and phenotypic correlations of behaviors in a natural population

**DOI:** 10.1007/s00265-015-2012-z

**Published:** 2015-09-28

**Authors:** László Zsolt Garamszegi, Gábor Markó, Eszter Szász, Sándor Zsebők, Manuel Azcárate, Gábor Herczeg, János Török

**Affiliations:** Department of Evolutionary Ecology, Estación Biológica de Doñana-CSIC, c/Americo Vespucio s/n, 41092 Seville, Spain; Behavioural Ecology Group, Department of Systematic Zoology , Eötvös Loránd University, Pázmány P. sétány 1/C, 1117 Budapest, Hungary; Department of Plant Pathology, Corvinus University of Budapest, Budapest Ménesi út 44, 1118 Budapest, Hungary; MTA-ELTE-MTM Ecology Research Group, Biological Institute, Eötvös Loránd University, Pázmány P. sétany 1/C, 1117 Budapest, Hungary; Grupo Ecología Evolutiva y de la Conducta, Estación Experimental de Zonas Áridas-CSIC, Ctra. de Sacramento s/n, La Cañada de San Urbano, 04120 Almería, Spain

**Keywords:** Boldness, Effect size, Flight initiation distance, Personality, Phenotypic correlation, Temperament

## Abstract

When mean behaviors correlate among individuals, they form behavioral syndromes. One way to understand the evolution of such a group-level phenomenon is to compare horizontally patterns of correlations among populations (or species) or follow longitudinally the same population over years in the light of parallel differences in the environment. We applied the longitudinal approach to 8-year field data and analyzed phenotypic correlations, and their within- and between-individual components, among three behaviors (novelty avoidance, aggression, and risk-taking) in male collared flycatchers, *Ficedula albicollis*, in a meta-analytic framework. The phenotypic correlation between novelty avoidance and aggression varied heterogeneously (it was positive in some years, while it was negative in other years), while the other pair-wise correlations were consistently positive over the study period. We investigated four potential socio-ecological factors, and found evidence that the among-year alterations in the demographic structure of the population (density, age composition) can be responsible for the heterogeneous effect sizes. Comparing within- and between-individual correlations across pairs of traits, we found that the correlation between aggression and risk-taking at the among-individual level was the strongest suggesting that this relationship has the highest potential to form a behavioral syndrome. Within-year repeatabilities varied among traits, but were systematically higher than between-year repeatabilities. Our study highlights on an empirical basis that there can be several biological and statistical reasons behind detecting a phenotypic correlation in a study, but only few of these imply that fixed behavioral syndromes are maintained in a natural population. In fact, some correlations seem to be shaped by environmental fluctuations.

## Introduction

A striking recognition of recent day’s evolutionary behavioral ecology is that, although one would expect individual animals to adaptively adjust each of their behaviors depending on the prevailing environmental conditions, apparently many behaviors cannot vary with unlimited flexibly and in isolation from others (Réale et al. 2007). Linked behaviors form behavioral syndromes, in which the non-independence of traits constrains the evolutionary trajectories that are available for particular behaviors (Dochtermann and Dingemanse 2013). A fundamental question is, therefore, why and how such syndromes are maintained over generations (Dall et al. 2004; Dingemanse and Wolf 2010; Wolf and Weissing 2010).

Behavioral syndromes can be defined as the between-individual correlation of functionally independent behaviors (Sih et al. 2004a, b; Dingemanse and Wolf 2010; Herczeg and Garamszegi 2012). Therefore, to study behavioral syndromes, it is inevitable to obtain repeated measures on the same behavior from the same individuals that allow discriminating between the within-individual and the between-individual correlations (Dingemanse et al. 2012; Garamszegi and Herczeg 2012; Brommer 2013; Dingemanse and Dochtermann 2013). The former type of correlation can emerge if correlative behavioral responses to the same environmental factor occur plastically at the within-individual level (e.g., within-individual correlations between exploration and aggression can develop if at low temperatures individuals are rather inactive, generally less aggressive, and less explorative, while at higher temperatures they become more aggressive and explorative). Only between-individual correlations reflect links between individual-specific attributes and are relevant for behavioral syndromes. Practically, if one collects a single measurement for each trait from each individual, correlations between behaviors will provide phenotypic correlations, which combine the between-individual and within-individual components with unknown magnitudes. Making inferences from such phenotypic correlations for behavioral syndromes (as done in many studies) relies on the assumption that within-individual variation is negligible (Brommer 2013), which is a strong interpretive step as behaviors are typically very plastic traits (Bell et al. 2009).

Given that behavioral syndromes translate into between-individual correlations (or phenotypic correlations as a surrogate), such a phenomenon is inevitably a population-specific attribute thus is manifested only at a higher group level. Therefore, one way to investigate how behavioral syndromes can evolve is to compare correlation structures across different populations or species that experience different selection regimes, and to determine the socio-ecological factors that generate differences in the strength and direction of these correlations (Conrad et al. 2011; Herczeg and Garamszegi 2012; Sih et al. 2012; Carvalho et al. 2013). Embracing such a framework based on groups of individuals as the unit of analysis, Bell (2005) and Dingemanse et al. (2007) investigated the activity-aggression-boldness syndrome in different populations of the three-spined stickleback (*Gasterosteus aculeatus*) that inhabit different selective environments and genetically differentiated from each other. They found that certain types of correlations are population–specific, which could have resulted from population-level adaptations to presence or absence of predation. Similar patterns concerning the population-specific correlations have also been described for other taxa (Scales et al. 2011; Bengston et al. 2014; Martins and Bhat 2014), but evidence at conflict with the between-population divergence of behavioral syndromes has also been reported (Brydges et al. 2008; Herczeg et al. 2009; Pruitt et al. 2010). At a wider scale, meta-analyses comparing a larger number of populations of different species also detected interspecific differences in behavioral syndromes as inferred from phenotypic correlations across individuals, which can be interpreted, at least in part, as the consequence of the dissimilarities in adaptation processes that species underwent during their phylogenetic history (Garamszegi et al. 2012a, 2013). However, the comparisons of entities that have been isolated over a phylogeographic time scale do not allow discriminating whether differences in the correlation structure that are observed among particular populations (or species) are the result of (i) long-term adaptation processes that generate genetic differentiation and that stably couples or uncouples behaviors, or (ii) phenotypic plasticity by which phenotypic correlations are enforced by the specific environments in which populations occur, or (iii) both.

An alternative approach to the horizontal comparison between populations/species would be to perform a longitudinal analysis of correlations of the same population over much smaller time scales. Monitoring concurrent changes in the environment would allow understanding how rapidly and unpredictably altering environmental components can affect the correlation structure of behaviors independently of processes due to genetic adaptation (e.g., Sinn et al. 2010; Kazama et al. 2012). In such a longitudinal framework, detected phenotypic correlations could vary among years (or other time scales) both for statistical and biological reasons. Statistically, detected correlations can be different because (i) between-individual correlations vary (i.e., due to differences in genetic or permanent environment correlations), because (ii) within-individual correlations vary, because (iii) correlations due to measurement error vary, or because (iv) the combinations of these vary among samples (Dingemanse et al. 2012; Garamszegi and Herczeg 2012; Dingemanse and Dochtermann 2013).

The biological reasons behind temporal alterations in the correlation structure can include processes due to phenotype-dependent selection and phenotypic plasticity. For example, yearly shifts in, e.g., predation pressure, food supply, or/and social constraints can impose differential selection pressures on the reproductive success or survival of different phenotypes (Dingemanse et al. 2004). As a consequence, the structure of the population will be affected in a way that the yearly samples of individuals will represent different genetic or permanent environment correlations. On the other hand, differences in phenotypic correlations can be attributed to differences in within-individual correlations if variation in environmental conditions makes individuals to change their behaviors from one reproductive event to the next (Bell and Sih 2007; Shimada et al. 2010; Sih et al. 2011; Dingemanse and Wolf 2013). Such phenotypic plasticity would allow fine adaptation at the individual–level, in which the prevailing environmental conditions elicit the most beneficial display from the individuals’ behavioral repertoire. These two extreme scenarios are certainly mixed in natural populations, as multiple biological processes can be in effect simultaneously for the same behavioral correlation, and processes due to both phenotype-dependent selection and phenotypic plasticity can be in action in parallel. To make it more complex, different mechanisms may be applied to different pairs of behaviors. Therefore, it would be desirable to obtain deeper insights from wild populations of animals in how behavioral correlations vary among years and to uncover the statistical and biological causes of such variations by partitioning the within- and between-individual correlations and also by identifying parallel changes in the socio-ecological environment.

When the purpose is to compare patterns of correlations between traits, the meta-analytic framework offers a powerful tool to obtain a quantitative summary over a suite of studies that provide information on different groups of individuals (Wilson and Lipsey 2000; Borenstein et al. 2009; Ellis 2010). Such an approach can estimate the overall strength and direction of any biological association in the form of an effect size by accounting for the underlying sample size, assess the degree of heterogeneity that arises among the findings of the source studies, and to statistically evaluate how methodological or biological factors shape such differences in the study results. One can borrow the meta-analytic methodology to deal with the among-year variation in a biological association that occurs within the same population, as different years can be treated as separate studies. This focus differs from that of the classical ecological application in that the former covers variation in short temporal scales, while the latter typically targets larger-scale variation across different populations/species that are separated by geographic distances; thus, the results have different biological implications. The benefit of applying the meta-analysis to the same system that is consistently studied by the same standards is that it is not loaded with heterogeneity due to methodology and publication bias (Kotiaho 2002).

Here, our goal was to uncover whether the phenotypic correlations that can be detected in certain years in a natural population are the result of long-term processes that generate stable links between different behaviors, or vary more sensitively, as a potential response to the prevailing environmental conditions. The former mechanism predicts that the strength and direction of the phenotypic correlations between repeatable behaviors are caused by between-individual correlations and remain consistent and similar across years. However, the latter scenario predicts considerable between-year variation in the correlation structure (that is potentially caused by within-individual correlations) if the environment also fluctuates. We tested these predictions in a Hungarian population of the collared flycatcher, *Ficedula albicollis*, in which we routinely monitor different behaviors in males (novelty avoidance, aggression, risk-taking) during courtship (e.g., Garamszegi et al. 2006, 2009, 2012b). We used field data from 8 years, in which we scored the focal behavioral traits upon the arrival of males from the wintering grounds to calculate phenotypic correlations. In 5 years, we also collected repeated measurements from the same individuals, which permitted us to calculate within- and between-individual correlations as well as repeatabilities in these seasons. Furthermore, we characterized among-year variation in some environmental factors by estimating year-specific predation pressure, mean daily temperature (potentially affecting the availability for food), density (potentially affecting the availability for breeding opportunities), and age composition. As an explorative, hypothesis-generating exercise, we related these environmental variables to among-year variation in correlation structures. Our investigations relied on a meta-analytic framework that enabled us to rigorously compare year-specific correlations among behavioral and ecological traits.

## Materials and methods

### General behavioral measurements to obtain phenotypic correlations

Our fieldwork for this study was carried out in a nest-box population of the collared flycatcher in the Pilis Mountains close to Budapest, Hungary (47°43′N, 19°01′E). In the breeding seasons 2007 to 2015, we applied non-invasive (i.e., without capturing individuals) methods to characterize three behavioral traits in males. From the expected date of the first birds returning from the wintering sites, we regularly visited the field site for newly arrived, unpaired males showing the typical courtship behavior on their territory during the most active morning period (usually between 6.00 to 12.00 h). Once these males were localized at a nest-box, we performed behavioral assays based on standardized protocols that have been described in detail and validated elsewhere (e.g., Garamszegi et al. 2006, 2009, 2012b). We excluded year 2008, as we assayed less than five males in that breeding season and did not screen all behaviors (Table 1). Here, we only provide information that is important for the interpretation of the results.

**Table 1:** Summary statistics for the three behavioral variables of males that were collected in eight breeding seasons in a Hungarian population of the collared flycatcher to study between-year variation in phenotypic correlations in a meta-analysis. Sample size, mean, and standard errors are based on the sample of males that were assayed for their behaviors at least once upon their arrival to the breeding ground. Due to the very low sample size, data for 2008 was not used further

Year	Novelty avoidance (latency to land in seconds)			Aggression (latency to fight in seconds)			Risk-taking (flight initiation distance in meters)		
	*N*	Mean	SE	*N*	Mean	SE	*N*	Mean	SE
2007	21	113.2	36.8	23	50.4	21.8	21	11.8	1.5
2008	2	121.5	154.5	0	–	–	3	10.0	5.5
2009	33	12.5	23.1	34	29.7	12.7	32	13.0	1.4
2010	28	108.5	27.5	31	50.3	17.2	31	14.0	1.2
2011	40	195.6	17.6	54	55.8	13.9	51	10.3	0.7
2012	17	201.1	26.9	25	92.3	24.4	22	13.5	1.9
2013	44	138.6	22.8	56	44.5	12.8	54	9.8	0.8
2014	45	119.1	18.3	53	40.0	11.7	52	12.6	1.1
2015	40	110.6	24.1	46	17.4	7.8	47	7.5	0.7

We first estimated novelty avoidance, defined as the latency needed to resume a key element of courtship activity in the presence of a novel object. We assessed baseline courtship activity by placing a caged stimulus female on the top of the nest-box and measuring the time interval between the male’s appearance on the territory (based on the conspicuous coloration and behavior of males, we assumed that we can spot them immediately when they arrive on the territory) and its first landing on the entrance hole of the nest-box (by this behavior, male flycatchers aim at eliciting a nest-box visit from the female). Then, we attached a novel object (white A6 sheet with small random drawings of variable colors) on the front side of the box and took the same measurements (if a male did not land in the presence of novelty, we recorded 301 s for this observation based on the duration of the assay). Novelty avoidance was calculated as a difference between the latency scores from the two situations, and is the inverse estimate of how individuals tolerate the presence of a novelty stimulus.

After the novelty avoidance test, we scored aggression by exposing the focal bird to a caged stimulus male, with which we stimulated aggressive response from the territory owner. To describe aggression, we timed the latency to the first attack (i.e., the first touch on the cage of the decoy), as elapsed since the appearance of the resident on the territory. Latency to fight predicts several other behavioral variables that describe aggression (Garamszegi et al. 2006). If the male did not attack, we assigned a score of 301 s (our observations lasted 5 min).

When the subject was localized touching the decoy’s cage and being engaged in a territorial dispute, or was observed on another frequently visited position (nest-box, nearby branch), we initiated our assessment of risk-taking by measuring flight initiation distance (FID, Blumstein 2003). The observer started to walk towards the focal bird until it noticed the presence of a potential predator and interrupted its current display. The observer continued walking if the resident returned to the decoy’s cage (or another focal position) within at least 1 min. This sequence was repeated until the resident bird did not return anymore to this reference position (each individual returned at least once). The closest distance between the decoy and the last standing point of the observer was measured as the number of steps of approximately 1 m to reflect flight initiation distance. By our approach, we aimed at eliminating the confounding effect of very aggressive males not noticing the approaching human (by allowing the focal male to return, we ascertained than it had noticed the observer).

We captured males after the behavioral assays with a conventional nest-box trap for identification and to perform standardized ringing protocols and measurements. We were unable to capture and subsequently identify some birds (95 out of 337) after the behavioral assays. We have previously shown that such between-individual variation in trappability is associated with the differences in the screened behaviors, and the elimination of non-captured birds from the sample introduces bias when assessing behavioral correlations (Garamszegi et al. 2009). Such tendencies showing that individuals displaying shy behaviors are generally more difficult to capture were also prevalent in the current data covering eight field seasons (novelty avoidance: *t*_268_ = 2.652, *P* = 0.008; aggression: *t*_320_ = 2.290, *P* = 0.022, risk-taking: *t*_311_ = 3.359, *P* < 0.001). Therefore, to avoid such bias and a considerable loss in sample size, we did not exclude unidentified males from our analyses. However, such a strategy may potentially lead to the risk of generating partially non-independent observations, as unidentified males may be repeatedly present in different samples. We assume that the problem posed by the partial non-independence of data should be minor, as based on the list of successfully ringed individuals we estimate that the chance of assaying an individual in 2 or more years is 7.7 % (due to the modest return rate of the species—<15 % in adult males—and the fact that we can only monitor the behavior of a subsample of the population in each year).

### Repeated behavioral measurements to estimate within- and between-individual correlations

In five field seasons (2009, 2011, 2013, 2014, 2015), we made efforts to relocate the birds that had been previously assayed upon their arrival to obtain subsequent behavioral measurements until they established pair bounds (birds when caught after the first set of assays were individually marked on their belly with unique combinations of three colors by water-resistant pens). By doing so, we were able to repeat the behavioral tests for about the half of the males (see Table 3 for exact sample sizes) on average 2.74 times (range, two to six occasions). We used these multiple measurements to differentiate statistically between the within-individual and the between-individual correlations within years (see below). We note that repeated measurements could only be acquired for males that had been captured successfully after the first assay; thus, we could not eliminate biases due to differences in trappability (and in the probability of re-sights) in this subsample of males. Therefore, caution is needed when comparing phenotypic correlations with within- and between-individual correlations, as these correspond to different samples (see more details below).

### Socio-ecological variables

We described each breeding season by four types of ecological variables at the population–level for each year. To characterize year-specific weather conditions, we estimated the mean of daily temperature observed over the period between 15th April and 15th May (when the birds arrive and form pairs, i.e., when we took the behavioral measurements), as measured at a nearby meteorological station and supplied to the NOAA’s National Climatic Data Center (ftp://ftp.ncdc.noaa.gov/pub/data/gsod). This indirect climatic variable appeared to be a strong predictor of the average temperature that could by obtained directly via a small meteorological station that operated for some years in our field station (*r* = 0.972, *N* = 12, *P* < 0.001). Furthermore, we have found a strong correlation between the mean daily temperature and the estimated caterpillar biomass (*r* = 0.853, *N* = 12, *P* < 0.001; caterpillar biomass was estimated by collecting and weighting the produced caterpillar frass in a standard way, see Török and Tóth 1988). Given that caterpillars are one of the main items on the flycatchers’ diet (Löhrl 1976), we could reasonably assume that our climatic variable was a good predictor of yearly food supply.

Predation rate in each year was estimated as the proportion of nests that were found fully or partially predated from the egg laying to the chick-feeding period (breeding efforts were monitored in each nest-box based on regular checks). The most typical predator of the species is the Pine Marten *Martes martes* that leaves clear signatures upon their activity (heavily disturbed nest material, remainings of the chicks, or incubating females on the top of the nest-box). Based on our long-term data, nest predation rate varies from 0 to 48 % among years, which mostly involved chick mortality. Given that such predation events occur *after* the behavioral assays, we assumed that, if it applies at all, the predation pressure estimated in 1 year during the period between egg laying and chick-feeding should only affect behavioral performance of males during the courtship period *in the next year*. Increase in predation rate in a given year can have considerable influence on several demographic parameters in the subsequent year thus rise differences in the composition of the population (for example, predation rate in 1 year determines the proportion of immigrant males in the next year: *r* = −0.721, *N* = 18 years, *P* < 0.001). Furthermore, the degree of predation can affect individual experience, which can determine risk-taking decisions during the future reproductive events. Therefore, we matched year-specific behavioral correlations with predation rate that corresponds to the previous year.

The degree of competition for nest-boxes among males due to density effects was determined by considering the number of potential breeding opportunities estimated from the number of available nest-boxes relative to the number of breeding pairs. For each year, we counted the total number of nest-boxes that were available for the collared flycatcher for breeding (i.e., the number of nest-boxes that were finally occupied by the collared flycatchers plus the number of empty boxes, i.e., that were left uninhabited by other hole nesting species that typically start breeding before flycatchers arrive). Relative density was then calculated as the number of breeding efforts of flycatchers/available nest-boxes. We further corrected this estimate for synchrony effects because the level of competition should be higher when most birds compete for resources at the same time. Therefore, we determined the time interval (in days) within which the 90 % of breeding efforts occurred and with which we further divided the above density index to express average competition per day.

Given that age may affect individual experience, we also characterized the age structure of the male population. Upon the ringing protocols (as well as through the binocular observations of non-captured individuals), we assigned males into juvenile and adult age categories based on the typical coloration of the wing (Svensson 1984). Then, age structure was calculated for each year as the number of juvenile individuals relative to the total number of individuals by using the sample of males that were assayed for their behaviors.

### General statistical approaches

**Fig. 1:**
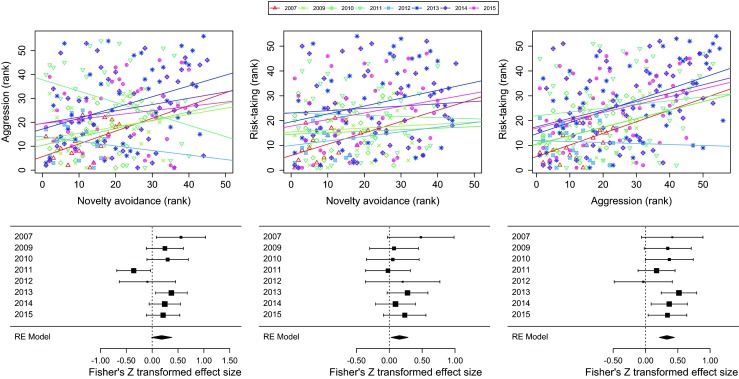
Year-specific phenotypic correlations among three behavioral traits of male collared flycatchers assayed during the courtship period of eight breeding seasons (2007–2015 with 2008 excluded). *Upper panels* show the pooled ranked raw data and the fitted regression lines using different colors and symbols for different years (individuals were ranked along their behaviors in each year in a way that lower ranks systematically signify bolder behaviors, i.e., lower novelty avoidance and higher aggression and risk-taking). *Lower panels* present the meta-analysis of the above data relying on years as unit of the analysis. *Black squares* represent year-specific effect size calculated from the corresponding Spearman rank correlation of traits, with a size proportional to the underlying sample size. *Horizontal error bars* represent the 95 % confidence intervals. *Diamonds* are the overall mean effect sizes, as calculated from a random-effect meta-analytic model over the whole 8-year sample, with a width showing 95 % confidence intervals. For exact sample sizes, see Table 1

All analyses were carried out in the R statistical environment (R Development Core Team 2015). Due to various constraints, information on some behaviors was not available in few cases causing slight variation in sample size both within and among years (see summary statistics for the yearly samples in Table 1). The distribution of novelty avoidance and aggression showed strong deviation from being normal even after trying various transformations. Therefore, to obtain standardized and comparable estimates for the strength of different relationships, we calculated Fisher’s *Z*-transformed Spearman rank correlations between the three behavioral variables in each year separately to describe group-level patterns (see also Dingemanse et al. 2007 for a similar approach in a between-population context). Previously (Garamszegi et al. 2008, 2009, 2012b), we have assessed the role of several potentially confounding factors (such as age and other attributes of males, territory quality, date of measurement, etc.) on these correlations and concluded that, except trappability, none of these seriously affected the focal relationships. Therefore, for simplicity, we did not consider additional covariates in this study and proceeded with raw correlations instead of building complex linear models with several covariates with minor effect. For illustrative purposes (Fig. 1), we present the rank-transformed raw data. The socio-ecological predictors that were calculated as proportions (predation rate, competition index, age structure) were square-root transformed.

To process repeated measurements on the same individuals and to calculate the within- and between-individual components of (co-)variances, we used univariate and bivariate mixed modeling (Dingemanse and Dochtermann 2013), available in the *MCMCglmm* (Hadfield 2010) R package. *MCMCglmm* relies on Markov Chain Monte Carlo processes for parameter estimation, for which we defined a relatively uninformative prior specification equivalent to an inverse gamma prior with shape and scale equal to 0.001 and with a belief parameter (nu) set to 1.002 (alternative prior settings, e.g., the use of the default of *MCMCglmm* do not affect qualitatively the results). Each model was run for 1.3 million iterations, sampling every 1000 (thinning interval) after discarding the first 300,000 (burnin). We checked models for convergence and mixing by examining the Gelman–Rubin statistics (Gelman and Rubin 1992; the potential scale reduction factor <1.1 for all parameters) among chains, and for autocorrelation within chains (Hadfield 2010). We also visually assessed the traces of all parameters for independence and consistency of the posterior distributions over iterations. To check the stability of results, each model was fitted at least three times, and we also verified if longer runs (i.e., based on 5 million iterations) gave similar results.

As for model definition, to assess the repeatability of traits, we created models assuming normally distributed errors, in which one of the behavioral variables was the response, the corresponding date of observation was the predictor (see the importance of controlling for date effects in Biro and Stamps 2015), and the identity of males was added as random effect term (only random intercept was modeled). From these models, we extracted the estimated variance components and calculated repeatability as the proportion of the between-individual variance relative to the total variance (Nakagawa and Schielzeth 2010; Dingemanse and Dochtermann 2013). The 95 % confidence interval of this metric was determined from the 95 % credibility interval of the posterior distribution of the *MCMCglmm* output. To calculate within-year repeatability, we repeated this procedure for each focal variable separately for each of the 5 years, in which multiple measurements for the same individuals were available. In the between-year context, we relied on males that were scored for their behaviors in more than 1 year over the 8-year period (we only used the first observation, i.e., the one that corresponds to the arrival date, from 1 year if repeated measurements were available within that year to control for potential date effects). To analyze patterns of variation in repeatability estimates, we used *t* tests, in which *t* values were calculated based on weighted means and weighted variances (where the weights are the years-specific sample sizes, i.e., the number of individuals, see Table 3). Accordingly, we applied weighted univariate *t* tests to check if the within-year repeatabilities of traits are systematically different from their between-year repeatabilities, and weighted paired *t* tests to compare within-year repeatabilities between pairs of traits.

For the assessment of within- and between-individual correlations, we constructed models (with normal error distributions) by using the pair-wise combination of behavioral traits as bivariate response and identity as random term. We used procedures described in Dingemanse and Dochtermann (2013) to obtain the two components of correlation for each relationship for each year. Above, we noted that our subsamples of males that have been used for this variance partition might be biased because we could only obtain multiple measurements for individuals that had been successfully captured and re-assayed. To evaluate the reliability of the estimates, we calculated the expected phenotypic correlations from them following the mathematical equation presented in Dingemanse and Dochtermann (2013), to which we also supplied the estimated within-year repeatabilities. Then we related these expected correlations to the phenotypic correlations that we actually observed in the entire dataset also including all non-captured males (note that within- and between-individual correlations could only be derived for birds that had been successfully re-assayed). We found a strong relationship between the two sets of estimates (*r* = 0.764, *N* = 15, *P* < 0.001) implying that the acquired within- and between-individual correlations are reliable.

### Meta-analyses

In a meta-analysis, first, the outcome of each study (yearly samples in the current context) is converted to a common currency so-called effect size, which is thus comparable across studies (see a comprehensive description about the method in Nakagawa and Santos 2012). Then, an overall effect size is calculated across studies, which is weighted by the precision of the study, with a confidence interval to reflect the precision of the estimate. We used the Fisher’s *Z*-transformed Spearman rank correlations as effect sizes, for which we derived confidence intervals based on their variance calculated as 1 / (*N* − 3), where *N* is the corresponding sample size (number of individuals). To calculate weighted mean effect sizes over the whole 8-year sample, we performed random-effect meta-analytic models assuming that each study year has its own effect size and allowing that they can be different from each other due to biological reasons. We particularly dealt with this degree of dissimilarity across findings by performing tests of heterogeneity (DerSimonian and Laird 1986). If we found evidence for such strong variance in effect sizes, we further examined if the detected heterogeneity can be attributed to the between-year variance in any socio-ecological factor by applying meta-regression (testing for the effect of moderators in a meta-analysis only makes sense, when the effect sizes truly vary across study samples). We relied on the package *metafor* (Viechtbauer 2010) for the meta-analytic procedures. For interpretations with regard to the magnitude of the effect, we followed the widely followed benchmarks from evolutionary ecology and other disciplines, in which untransformed *r* ≈ 0.1 is a small effect, *r* ≈ 0.3 is a moderate effect, and *r* ≈ 0.5 is a strong effect (Cohen 1988; Møller and Jennions 2002).

## Results

### Phenotypic correlations

The upper panels of Fig. 1 show the relationships as estimated from phenotypic correlations between the ranks of the three behavioral traits separately for each of the 8 years (note that ranks corresponding to latency scores or distances are all inverse estimates of exploration, aggression, and risk–taking, respectively; thus, positive correlations between ranks systematically imply that bolder individuals in one test are also bold in the other test). The visual inspection of these graphs suggests that although there seems to be a general tendency for a positive relationship between behaviors across individuals, there is also considerable variation among pairs of traits and years. In fact, in some years, some relationships can turn negative (e.g., aggression and novelty avoidance in 2011).

When entering these correlations as effect sizes into a meta-analysis (lower panels of Fig. 1), we found that mean effect size for the relationship between novelty avoidance and aggression cannot be differentiated statistically from zero (untransformed *r* = 0.182, CI_95%_ = −0.011/0.361, *N* = 264, *P* = 0.065). The other two relationships were generally significant and positive (novelty avoidance and risk-taking: untransformed *r* = 0.155, CI_95%_ = 0.027/0.278, *N* = 255, *P* = 0.018; aggression and risk-taking: untransformed *r* = 0.320, CI_95%_ = 0.211/0.420, *N* = 307, *P* < 0.001). A comparison of the effect sizes for the two significantly positive relationships yielded a statistically distinguishable, twofold difference in their magnitude (*z* = 2.06, *P* = 0.039). Another remarkable difference in the between-year patterns of phenotypic correlations of behaviors was that the relationship between novelty avoidance and aggression was heterogeneous (including both positive and negative correlations) among study years (*I*^2^ = 56.01 %, *Q* = 15.95, *df* = 7, *P* = 0.026), but we could not derive such evidence for the other two relationships (novelty avoidance and risk-taking: *I*^2^ = 0 %, *Q* = 4.058, *df* = 7, *P* = 0.773; aggression and risk-taking: *I*^2^ = 0 %, *Q* = 5.746, *df* = 7, *P* = 0.570).

### Within- and between-individual correlations

**Fig. 2:**
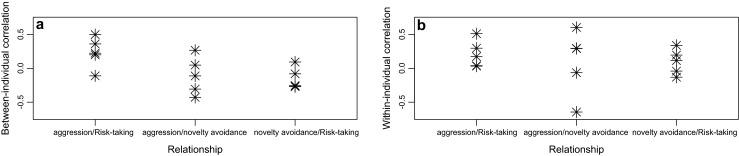
The effect of the type of correlation on within- and between-individual correlations. *Asterisks* are year-specific point estimates of effect sizes

We performed some simple analyses to explore patterns of among-year variation in the within- and between-individual correlations for those five study years when repeated measurements for the same individuals were available. When pooling correlations across years and the type of relationships, we found that year effects did not raise any heterogeneity either in the between-individual correlation effect sizes (*Q* = 0.212, *df* = 1, *P* = 0.645) or in the within-individual correlation effect sizes (*Q* = 0.285, *df* = 1, *P* = 0.594). However, we discovered that the type of the relationship was a significant predictor of the between-individual correlations, as the relationship between aggression and risk-taking was generally stronger and more consistent than the other relationships (*Q* = 9.826, *df* = 1, *P* = 0.007, Fig. 2a). Similar conclusions could not be made for the within-individual components (*Q* = 0.373, *df* = 1, *P* = 0.830; Fig. 2b). However, it is noteworthy that the among-year variance in the within-individual correlation for the novelty avoidance/aggression relationship is the highest. A visual inspection of the data revealed that the between- or within-individual correlations covered similar ranges mostly in the positive direction (Fig. 2), which were also comparable with the variation in the phenotypic correlations (Fig. 1).

### The role of ecological factors

**Fig. 3:**
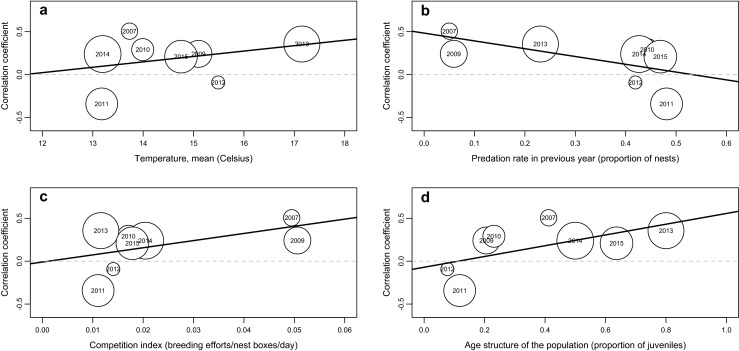
Meta-regressions demonstrating the effects of four socio-ecological variables on the phenotypic correlation between novelty avoidance and aggression in male collared flycatchers. *Each circle* represents a correlation that was observed in the designated year with a size that is proportional to the underlying sample size (see Table 1). For the definition and calculation of the ecological predictors, see the “Materials and Methods” section. *Solid lines* are the regression lines as were derived from the underlying meta-analyses using the given socio-ecological variable as mediator. *Dashed grey lines* represent *r* = 0 correlations and are shown for guidance

We examined if between-year variance in certain ecological factors can cause heterogeneity in the detected within-year patterns of phenotypic correlations between novelty avoidance and aggression (we explored the role of ecological predictors only for this particular correlation because only this covered a considerable variation among year-specific effect sizes that could be explained by a moderator variable). Corresponding meta-regressions revealed that the age composition of the population significantly affected the correlation between the two behaviors when they were entered in a pair-wise fashion in the model (Table 2 and Fig. 3). However, when we included the moderators simultaneously into the same model, we found that both demographic parameters (competition index and age structure) became significant predictors (Table 2).

**Table 2:** The effects of four moderator variables on year-specific phenotypic correlations between novelty avoidance and aggression when assessed via meta-regression approaches. On the left side, statistics are given for the cases when moderator variables were tested one by one in different meta-analytic models. On the right side, the effects correspond to a single multivariate regression model, in which the moderators were entered simultaneously (predation pressure was not included in this multivariate model because it strongly correlated with competition index: *r* = −0.887, *N* = 9, *P* = 0.001). Lower and upper 95 % confidence intervals for the correlation are given in brackets

moderator	Pair-wise model			Multivariate model		
	*Q* (*df* = 1)	*r*	*P*	*Q* (*df*=3)	*r*	*P*
Mean daily temperature	0.692	0.322 (−0.418/0.752)	0.406		0.224 (−0.600/0.771)	0.645
Predation pressure in previous year	2.452	−0.539 (−0.821/0.159)	0.117		Not included	
Competition index	1.833	0.484 (−0.240/0.804)	0.176		0.746 (0.138/0.903)	0.025
Age structure	4.671	0.662 (0.082/0.860)	0.031		0.767 (0.221/0.909)	0.016
Full model				12.353		0.006

### Within- and between-year repeatabilities

The repeatability of behaviors in different contexts is summarized in Table 3. Focusing on the within-year patterns, repeatability for risk-taking appeared to be consistently higher than for the other two traits (weighted paired *t* tests, novelty avoidance vs. aggression: *t*_4_ = 0.784, *P* = 0.477; novelty avoidance vs. risk-taking: *t*_4_ = −2.532, *P* = 0.065; aggression vs. risk-taking: *t*_4_ = −2.964, *P* = 0.041). Furthermore, there was a systematic tendency for within-year repeatabilities being higher than between-year repeatabilities (weighted one-sample *t* tests, novelty avoidance: *t*_4_ = 2.352, *P* = 0.078; aggression: *t*_4_ = 1.807, *P* = 0.145; risk-taking: *t*_4_ = 2.564, *P* = 0.062; Fisher’s combined significance for the three tests: *P* = 0.024).

**Table 3:** Within- and between-year repeatabilities of traits. Within-year repeatabilities are given for 5 years and are based on individuals that were successfully scored for their behaviors at least two times during the courtship period of the same breeding season. The corresponding samples were also used to calculate within- and between-individual correlations (see Fig. 3). Between-year repeatabilities originate from the entire database covering the 8-year period and were calculated by using males that were tested in at least two different breeding seasons (but only the first measurement was taken from 1 year). Lower and upper 95 % confidence intervals are given in brackets

Year	Novelty avoidance (latency to land)		Aggression (latency to fight)		Risk-taking (flight initiation distance)	
	*N*	Repeatability	*N*	Repeatability	*N*	Repeatability
2009	27	0.449 (0.003/0.774)	27	0.345 (0.032/0.631)	26	0.652 (0.406/0.837)
2011	16	0.047 (0.000/0.482)	16	0.037 (0.001/0.218)	16	0.116 (0.011/0.432)
2013	25	0.235 (0.000/0.629)	28	0.061 (0.002/0.232)	28	0.414 (0.153/0.646)
2014	16	0.046 (0.000/0.403)	17	0.185 (0.002/0.565)	17	0.517 (0.070/0.820)
2015	18	0.104 (0.000/0.525)	19	0.147 (0.002/0.535)	19	0.109 (0.006/0.402)
Between-year	19	0.021 (0.000/0.251)	21	0.058 (0.001/0.314)	21	0.117 (0.009/0.450)

## Discussion

Here, we studied among-year variation in repeatability and different types of correlations between three behavioral traits in collared flycatcher males from a free-living population. The major findings were the followings. First, we found that phenotypic correlations for the novelty avoidance/risk-taking and for the aggression/risk-taking relationships remained systematically positive across years, while for the novelty avoidance/aggression relationship, they varied considerably between years in terms of both magnitude and sign. Second, we were able to demonstrate that such heterogeneous variation in effect sizes for the latter relationship could be mediated by the among-year alterations in the studied demographic factors determining the level of competition for breeding opportunities and age composition of the population. Third, within-year repeatability of traits varied among the assayed behaviors (it was the highest for risk-taking) and tended to be considerably higher than their between-year repeatability. Finally, we observed that the within-year between-individual correlations differed among the considered pairs of traits, as the aggression/risk-taking relationship was consistently stronger than the other relationships.

The difference in the mean and variance in effect sizes across pairs of behaviors may question the existence of an universally applicable explanation for phenotypic correlations among repeatable behavioral traits that are often interpreted as evidence for behavioral syndromes (Dingemanse et al. 2012; Garamszegi et al. 2012a; Brommer 2013). The novelty avoidance/aggression and novelty avoidance/risk-taking relationships can be characterized by a similarly small overall effect size (*r* < 0.2), but the former includes much larger heterogeneity in terms of both magnitude and direction of effect sizes (which causes that the mean effect size cannot be statistically differentiated from zero in the current sample) than the latter. However, when we focus on phenotypic correlations that homogeneously appear positive in different years, we can still observe twofold differences in their means. In fact, the aggression/risk-taking relationship reached a magnitude that represents moderate effect size, while the novelty avoidance/risk-taking relationship could only be interpreted as being a small effect size. Furthermore, the largest phenotypic correlation between aggression and risk-taking was accompanied by the largest between-individual correlation indicating that each pair-wise relationship was loaded with different within- and between-individual components. Therefore, even if the studied phenotypic correlations appear positive in overall, the differences in their strengths and the heterogeneity they cover should signify differences in their biological meaning. We infer that only some of these correlations fulfill criteria for behavioral syndromes.

Behavioral syndromes can be maintained in a population if there are rigid genetic, maternal, or early environmental effects that build up developmental or physiological constraints that finally keep behaviors linked together over longer evolutionary time scales (Sih et al. 2004a, b; Bell 2005; Dochtermann and Dingemanse 2013). Such mechanisms would raise stable between-individual correlations that are independent of the short-term and unpredictable changes in the environment, and could be potentially responsible for the detected patterns in association aggression/risk-taking relationship in the among-year context. In a previous study focusing on the proximate effects of two functionally different genes (dopamine receptor *D4* gene and the major histocompatibility complex), we found that flight initiation distance was the variable that depicts the strongest relationships with the genetic profile at these regions (Garamszegi et al. 2014, 2015). These findings may imply that observed among-individual variation in this behavioral phenotype is mediated by genetic differences among individuals. The current observation that within-year repeatability is the highest for this behavior is also in line with this interpretation. We also note that between-year repeatability for this trait, although it was small, was also the highest and could be differentiated from zero suggesting that between-individual differences in risk-taking remain preserved, at least to some degree, on a longer time scale.

The heterogeneous phenotypic correlation between novelty avoidance and aggression, on the other hand, may have resulted from year to year changes in either the between-individual or in the within-individual component. Under this scenario, detected syndromes would not be stabilized by strict mechanistic constraints but would be sensitive to fluctuations in the environment (Bell 2005, see also Fig. 2 and Table 2 in the current study) through plasticity or phenotype-dependent selection (Bell and Sih 2007; David et al. 2014). Accordingly, between-individual correlations for the same relationships could vary among years if, as a consequence of a socio-ecological factor, individuals alter their behavioral phenotypes in a between-year context, even though they maintain individual-specific correlation structures within the same breeding season. For example, one can imagine that trait combinations that are expressed in a given breeding season were shaped by experience early in that season/previous winter but are reshuffled in the next year when new information about the socio-ecological conditions is gathered. Given that (i) our between-individual correlations concern with the within-season context and does not say anything about between-individual correlations on a longer time scales, and that (ii) the between-year repeatability of traits was generally low, between-year changes in the between-individual correlation remains a plausible explanation for the results in association with the novelty avoidance/aggression relationship. If this applies, we can preclude that strong genetic (such as in Dochtermann 2011) or long-lasting early environmental effects (such as in Sweeney et al. 2013; Bengston et al. 2014; Urszán et al. 2015) shape the between-individual correlations. On the other hand, the mediator effects of the demographic parameters (age-structure and degree of competition) may imply that individual experience and/or year-specific adjustments to the available breeding opportunities play more important roles. Alternatively, we can also imagine that among-year variation in the correlation patterns emerged not because of between-year adjustments within individuals, but because of the yearly shifts in the composition of individuals in the population. Therefore, along the sequence of the study, we would have sampled different groups of individuals that could be characterized by different between-individual correlations, which is also a scenario to be considered given the minimal overlap between our yearly samples. This could have occurred, for example, if certain environmental factors had an effect on the survival, reproductive output, and/or dispersal of individuals (Bell and Sih 2007; Logue et al. 2009), and fluctuations in the age-structure and levels of competition have reflected such year-specific phenotype-dependent selection pressures.

We cannot exclude the possibility that short-term within-individual effects mediate phenotypic correlations at least in some years (see theory in the “Introduction,” empirical examples can be found in Araya-Ajoy and Dingemanse 2014; Brommer et al. 2014; Fresneau et al. 2014; Dosmann et al. 2015). For example, the statistically significant negative relationship between novelty avoidance and aggression that appeared in 2011 had a very strong within-individual component (Fig. 3). Between-year differences in the within-individual correlations can occur, for instance, if particular socio-ecological factors affect the within-season plasticity of behaviors in a year-specific way. Hence, there might be years (e.g., when there are many competitors in the population that is also shifted toward juvenile-biased age structure, Fig. 2) when specific within-individual correlations are enforced leading to that if an individual changes its level of novelty avoidance due to some reasons it also alters its level of aggression in the same direction. In another year, such linked plastic responses may be relaxed or even go in the opposite direction resulting in the situations of no or negative within-individual correlation between the same traits.

We must note that our study has certain limitations, thus certain interpretations should be made with caution. The most important constraints arise from the available sample size. First, although we have assayed more than 300 individuals altogether (Table 1), our framework relied on year-specific focal units (correlation structures) that inherently limits sample size to *N* = 8. Meta-analyses can powerfully exploit such samples by accounting for within-year sample sizes, but the effect of particular years remains influential, and the estimated effects all correspond to very broad confidence intervals. Therefore, we cannot reject the hypothesis that we were unable to deliver statistical evidence for weaker effects that remained non-significant in the current study, or that the inclusion of additional years with influential effects to the analyses can change some of the results. Second, we also relied on modest sample size for the partition of variances and correlations into the within- and between-individual component. We could use two to six within-individual repeats for these estimations, which also raises statistical issues about precision and bias (Martin et al. 2011; Garamszegi and Herczeg 2012; van de Pol 2012; Dingemanse and Dochtermann 2013). At least, based on the derived within- and between-individual components, we were able to reconstruct the detected phenotypic correlations and delivered biologically meaningful results suggesting that our estimates were reliable. Third, we should also consider that some of the detected heterogeneities were mediated by variance in measurement errors and not by variance in a biological predictor. In any case, we believe that our study can be definitely expanded to alleviate the above limitations.

In summary, our pioneer effort focusing on the temporal variation in the correlation structure of behaviors brings attention to the often-neglected phenomenon that finding a correlation between phenotypes in a given study year does not necessarily mean that the same correlation exists in another year. For the study of behavioral syndromes, this implies that finding non-significant correlation between behavioral traits in a narrow study period does not necessarily preclude that syndromes can be formed and detected in other environmental circumstances and based on a larger sample. Furthermore, we also highlight on an empirical basis that variation in phenotypic correlations can be due to variation in both the within-individual and between-individual components. This emphasizes the possibility that different biological explanations are responsible for different phenotypic correlations that are detected in a study system, and only few of these are in conformity with the definition for behavioral syndromes. We suggest that at least some of the phenotypic correlations appearing in wild animals are ecologically or contextually enhanced phenomena that may supersede genetically enforced rules and render within- and/or between-individual correlations spatially and temporally structured. Future research would benefit from the identification of additional socio-ecological factors that mediate long-term among-year variance in the correlation between pairs of behaviors, and also from deeper studies on within- and between-individual correlations that are manifested on longer time scales (e.g., among years). Our meta-analytic framework can be fruitfully applied along these directions, and it can be easily accommodated to deal with questions in relation to changes in the correlation structure in space and time.

In a wider context, our results point to the importance of the replicability and generalization of findings. Studies are very rare that are able to demonstrate that a relationship that is detected in 1 year is also persistent in other years when environmental condition are different (van Noordwijk 1998). To make strong conclusions about general patterns from field studies is only straightforward if the same findings can be delivered in a set of independent studies (coming from different years or populations), and a statistical summary over these repetitions unanimously reveals evidence for homogeneous patters. When heterogeneity is detected, it is of scientific interest to identify the sources of such heterogeneity (that can be either ecological or methodological).

## References

[CR1] Araya-Ajoy YG, Dingemanse NJ (2014). Characterizing behavioural ‘characters’: an evolutionary framework. Proc R Soc B.

[CR2] Bell AM (2005). Behavioural differences between individuals and two populations of stickleback (*Gasterosteus aculeatus*). J Evol Biol.

[CR3] Bell AM, Sih A (2007). Exposure to predation generates personality in threespined sticklebacks (*Gasterosteus aculeatus*). Ecol Lett.

[CR4] Bell AM, Hankison SJ, Laskowski KL (2009). The repeatability of behaviour: a meta-analysis. Anim Behav.

[CR5] Bengston SE, Pruitt JN, Riechert SE (2014). Differences in environmental enrichment generate contrasting behavioural syndromes in a basal spider lineage. Anim Behav.

[CR6] Biro PA, Stamps JA (2015). Using repeatability to study physiological and behavioural traits: ignore time-related change at your peril. Anim Behav.

[CR7] Blumstein DT (2003). Flight-initiation distance in birds is dependent on intruder starting distance. J Wildl Manag.

[CR8] Borenstein M, Hedges LV, Higgins JPT, Rothstein HR (2009). Introduction to meta-analysis.

[CR9] Brommer JE (2013). On between-individual and residual (co)variances in the study of animal personality: are you willing to take the “individual gambit”?. Behav Ecol Sociobiol.

[CR10] Brommer JE, Karell P, Ahola K, Karstinen T (2014). Residual correlations, and not individual properties, determine a nest defense boldness syndrome. Behav Ecol.

[CR11] Brydges NM, Colegrave N, Heathcote RJP, Braithwaite VA (2008). Habitat stability and predation pressure affect temperament behaviours in populations of three-spined sticklebacks. J Anim Ecol.

[CR12] Carvalho CF, Leitao AV, Funghi C, Batalha HR, Reis S, Mota PG, Lopes RJ, Cardoso GC (2013). Personality traits are related to ecology across a biological invasion. Behav Ecol.

[CR13] Cohen J (1988). Statistical power analysis for the behavioural sciences.

[CR14] Conrad JL, Weinersmith KL, Brodin T, Saltz JB, Sih A (2011). Behavioural syndromes in fishes: a review with implications for ecology and fisheries management. J Fish Biol.

[CR15] Dall SRX, Houston AI, McNamara JM (2004). The behavioural ecology of personality: consistent individual differences from an adaptive perspective. Ecol Lett.

[CR16] David M, Salignon M, Perrot-Minnot M-J (2014). Shaping the antipredator strategy: flexibility, consistency, and behavioral correlations under varying predation threat. Behav Ecol.

[CR17] DerSimonian R, Laird NM (1986). Meta-analysis in clinical trials. Control Clin Trials.

[CR18] Dingemanse NJ, Dochtermann NA (2013). Quantifying individual variation in behaviour: mixed-effect modelling approaches. J Anim Ecol.

[CR19] Dingemanse NJ, Wolf M (2010). A review of recent models for adaptive personality differences. Philos T Roy Soc B.

[CR20] Dingemanse NJ, Wolf M (2013). Between-individual differences in behavioural plasticity within populations: causes and consequences. Anim Behav.

[CR21] Dingemanse NJ, Both C, Drent PJ, Tinbergen JM (2004). Fitness consequences of avian personalities in a fluctuating environment. Proc R Soc Lond B.

[CR22] Dingemanse NJ, Wright J, Kazem AJN, Thomas DK, Hickling R, Dawnay N (2007). Behavioural syndromes differ predictably between 12 populations of three-spined stickleback. J Anim Ecol.

[CR23] Dingemanse NJ, Dochtermann NA, Nakagawa S (2012). Defining behavioural syndromes and the role of “syndrome deviation” in understanding their evolution. Behav Ecol Sociobiol.

[CR24] Dochtermann NA (2011). Testing Cheverud’s conjecture for behavioral correlations and behavioral syndromes. Evolution.

[CR25] Dochtermann NA, Dingemanse NJ (2013). Behavioral syndromes as evolutionary constraints. Behav Ecol.

[CR26] Dosmann AJ, Brooks KC, Mateo JM (2015). Within-individual correlations reveal link between a behavioral syndrome, condition, and cortisol in free-ranging Belding’s ground squirrels. Ethology.

[CR27] Ellis PD (2010). The essential guide to effect sizes: an introduction to statistical power, meta-analysis and the interpretation of research results.

[CR28] Fresneau N, Kluen E, Brommer JE (2014). A sex-specific behavioral syndrome in a wild passerine. Behav Ecol.

[CR29] Garamszegi LZ, Herczeg G (2012). Behavioural syndromes, syndrome deviation and the within- and between-individual components of phenotypic correlations: when reality does not meet statistics. Behav Ecol Sociobiol.

[CR30] Garamszegi LZ, Rosivall B, Hegyi G, Szöllõsi E, Török J, Eens M (2006). Determinants of male territorial behavior in a Hungarian collared flycatcher population: plumage traits of residents and challengers. Behav Ecol Sociobiol.

[CR31] Garamszegi LZ, Eens M, Török J (2008). Birds reveal their personality when singing. PLoS ONE.

[CR32] Garamszegi LZ, Eens M, Török J (2009). Behavioural syndromes and trappability in free-living collared flycatchers, *Ficedula albicollis*. Anim Behav.

[CR33] Garamszegi LZ, Markó G, Herczeg G (2012). A meta-analysis of correlated behaviours with implications for behavioural syndromes: mean effect size, publication bias, phylogenetic effects and the role of mediator variables. Evol Ecol.

[CR34] Garamszegi LZ, Rosivall B, Rettenbacher S, Markó G, Zsebők S, Szöllősi E, Eens M, Potti J, Török J (2012). Corticosterone, avoidance of novelty, risk-taking and aggression in a wild bird: no evidence for pleiotropic effects. Ethology.

[CR35] Garamszegi LZ, Markó G, Herczeg G (2013). A meta-analysis of correlated behaviors with implications for behavioral syndromes: relationships between particular behavioral traits. Behav Ecol.

[CR36] Garamszegi LZ, Mueller JC, Markó G, Szász E, Zsebők S, Herczeg G, Eens M, Török J (2014). The relationship between DRD4 polymorphism and phenotypic correlations of behaviors in the collared flycatcher. Ecol Evol.

[CR37] Garamszegi LZ, Zagalska-Neubauer M, Canal D, Markó G, Szász E, Zsebők S, Szöllősi E, Herczeg G, Török J (2015). Malaria parasites, immune challenge, MHC variability, and predator avoidance in a passerine bird. Behav Ecol.

[CR38] Gelman A, Rubin DB (1992). Inference from iterative simulation using multiple sequences. Stat Sci.

[CR39] Hadfield JD (2010). MCMC methods for multi-response generalized linear mixed models: the MCMCglmm R package. J Stat Softw.

[CR40] Herczeg G, Garamszegi LZ (2012). Individual deviation from behavioural correlations: a simple approach to study the evolution of behavioural syndromes. Behav Ecol Sociobiol.

[CR41] Herczeg G, Gonda A, Merila J (2009). Predation mediated population divergence in complex behaviour of nine-spine stickleback (*Pungitius pungitius*). J Evol Biol.

[CR42] Kazama K, Niizuma Y, Watanuki Y (2012). Consistent individual variations in aggressiveness and a behavioral syndrome across breeding contexts in different environments in the Black-tailed Gull. J Ethol.

[CR43] Kotiaho JS (2002). Meta-analysis, can it ever fail?. Oikos.

[CR44] Logue DM, Mishra S, McCaffrey D, Ball D, Cade WH (2009). A behavioral syndrome linking courtship behavior toward males and females predicts reproductive success from a single mating in the hissing cockroach, *Gromphadorhina portentosa*. Behav Ecol.

[CR45] Löhrl H (1976). Studies of less familiar birds. 179. Collared flycatcher. Brit Birds.

[CR46] Martin JGA, Nussey DH, Wilson AJ, Reale D (2011). Measuring individual differences in reaction norms in field and experimental studies: a power analysis of random regression models. Methods Ecol Evol.

[CR47] Martins EP, Bhat A (2014). Population-level personalities in zebrafish: aggression-boldness across but not within populations. Behav Ecol.

[CR48] Møller AP, Jennions MD (2002). How much variance can be explained by ecologists and evolutionary biologists. Oecologia.

[CR49] Nakagawa S, Santos ESA (2012). Methodological issues and advances in biological meta-analysis. Evol Ecol.

[CR50] Nakagawa S, Schielzeth H (2010). Repeatability for Gaussian and non-Gaussian data: a practical guide for biologists. Biol Rev.

[CR51] Pruitt JN, Riechert SE, Iturralde G, Vega M, Fitzpatrick BM, Avilés L (2010). Population differences in behaviour are explained by shared within-population trait correlations. J Evol Biol.

[CR52] R Development Core Team (2015) R: a language and environment for statistical computing. R Foundation for Statistical Computing, Vienna, Austria, http://www.R-project.org

[CR53] Réale D, Reader SM, Sol D, McDougall PT, Dingemanse NJ (2007). Integrating animal temperament within ecology and evolution. Biol Rev.

[CR54] Scales J, Hyman J, Hughes M (2011). Behavioral syndromes break down in urban song sparrow populations. Ethology.

[CR55] Shimada M, Ishii Y, Shibao H (2010). Rapid adaptation: a new dimension for evolutionary perspectives in ecology. Popul Ecol.

[CR56] Sih A, Bell A, Johnson JC (2004). Behavioral syndromes: an ecological and evolutionary overview. Trends Ecol Evol.

[CR57] Sih A, Bell AM, Johnson JC, Ziemba RE (2004). Behavioral syndromes: an integrative overview. Q Rev Biol.

[CR58] Sih A, Ferrari MCO, Harris DJ (2011). Evolution and behavioural responses to human-induced rapid environmental change. Evol Appl.

[CR59] Sih A, Cote J, Evans M, Fogarty S, Pruitt J (2012). Ecological implications of behavioural syndromes. Ecol Lett.

[CR60] Sinn DL, Moltschaniwskyj NA, Wapstra E, Dall SRX (2010). Are behavioral syndromes invariant? Spatiotemporal variation in shy/bold behavior in squid. Behav Ecol Sociobiol.

[CR61] Svensson L (1984). Identification guide to European passerines.

[CR62] Sweeney K, Gadd RDH, Hess ZL (2013). Assessing the effects of rearing environment, natural selection, and developmental stage on the emergence of a behavioral syndrome. Ethology.

[CR63] Török J, Tóth L (1988). Density dependence in reproduction in the collared flycatcher (*Ficedula albicollis*) at high population levels. J Anim Ecol.

[CR64] Urszán TJ, Török J, Hettyey A, Garamszegi LZ, Herczeg G (2015). Behavioural consistency and life history of *Rana dalmatina* tadpoles. Oecologia.

[CR65] van de Pol M (2012). Quantifying individual variation in reaction norms: how study design affects the accuracy, precision and power of random regression models. Methods Ecol Evol.

[CR66] van Noordwijk A (1998). The absence of evidence and the evidence for an absence. Acta Oecol.

[CR67] Viechtbauer W (2010). Conducting meta-analyses in R with the metafor package. J Stat Softw.

[CR68] Wilson DB, Lipsey MW (2000). Practical meta-analysis.

[CR69] Wolf M, Weissing FJ (2010). An explanatory framework for adaptive personality differences. Philos T Roy Soc Lond B.

